# TAK-242 ameliorates contact dermatitis exacerbated by IL-36 receptor antagonist deficiency

**DOI:** 10.1038/s41598-020-57550-5

**Published:** 2020-01-20

**Authors:** Hidehiko Fukushima, Yohei Iwata, Soichiro Watanabe, Kenta Saito, Yoshihito Tanaka, Yurie Hasegawa, Masashi Akiyama, Kazumitsu Sugiura

**Affiliations:** 1 Department of Dermatology, Fujita Health University School of Medicine, 1-98 Dengakugakubo, Kutsukake-cho, Toyoake, Aichi 470-1192 Japan; 2 Department of Dermatology, Nagoya University Graduate School of Medicine, 65 Tsurumai-cho, Showa-ku, Nagoya, Aichi 466-8550 Japan

**Keywords:** Biophysics, Immunology, Diseases

## Abstract

Loss-of-function mutations in *IL36RN* cause generalized pustular psoriasis (GPP), which is characterized by neutrophil-infiltrated lesions. Neutrophils are important during contact hypersensitivity in mice. However, it has never been determined whether interleukin-36 receptor antagonist (IL-36Ra) deficiency is an exacerbating factor in contact dermatitis. We examined whether a loss-of-function *IL36RN* mutation exacerbates contact dermatitis and evaluated the changes in contact dermatitis-related cytokines. Wild-type and *Il36rn*^−/−^ mice were treated with 1-fluoro-2,4-dinitorobenzene (DNFB) and evaluated for ear thickness, histopathological features, numbers of infiltrated neutrophils, and numbers of CD4 + and CD8 + T cells. Furthermore, mRNA levels of contact dermatitis-related cytokines were measured by real-time polymerase chain reaction, and effects of TAK-242, a toll-like receptor 4 (TLR4) inhibitor, on the contact hypersensitivity (CHS) response were evaluated. We found that the ear thickness, cytokine expression, and neutrophil infiltration significantly increased in *Il36rn*^−/−^ mice compared with that in wild-type mice. TAK-242 alleviated CHS and prevented neutrophil infiltration, cytokine expression, and ear thickening in *Il36rn*^−/−^ mice. These data indicate that *Il36rn*^−/−^ mutations are an exacerbating factor for CHS and that TAK-242 can reduce the inflammatory responses that are associated with the CHS response.

## Introduction

Homozygous or compound heterozygous *IL36RN* gene mutations underlie the pathogenesis of psoriasis-related pustular eruptions, including generalised pustular psoriasis, palmoplantar pustular psoriasis, acrodermatitis continua of Hallopeau, and acute generalised exanthematous pustular eruptions^1^. Loss-of-function mutations in *IL36RN* define a recessively inherited autoinflammatory disease called “deficiency of interleukin-36 receptor antagonist (IL-36Ra)” (DITRA), which is a type of autoinflammatory keratinization disease^2–7^. Previously, we generated *Il36rn*^−/−^ mice and established a DITRA murine model^8^. The *IL36RN* gene encodes IL-36Ra, a protein in the IL-1 cytokine family responsible for the tight regulation of IL-36 signalling. The IL-36 pathway is activated when one of the three IL-36 agonists (IL-36α, β, and γ) binds to their common but specific receptor interleukin 1 receptor-related protein 2 (IL-1Rrp2), which recruits the co-receptor, IL-1 receptor accessory protein (IL-1RacP), and triggers downstream activation of NF-κB and MAPK kinase signalling pathways to ultimately enhance transcription and release of pro-inflammatory cytokines^9,10^ that initiate the recruitment of inflammatory cells, including neutrophils, T cells, and myeloid dendric cells, in the skin. Abnormal IL-36 receptor (IL-36R) signalling results in transient skin inflammation characterised by acanthosis, hyperkeratosis, and neutrophil-dominant mixed-cell infiltration^11–13^.

According to the Human Genetic Variation Database, two *IL36RN* founder mutations (c.28 C > T (p.Arg10X) and c.115 + 6 T > C (p.ArgfsX1)) are found in just under 2% of the Japanese population^14^. Thus, many Japanese have *IL36RN* mutations that could be the pathogenic factor for many diseases, including generalised pustular psoriasis. Recent studies have found that neutrophils play an important role in the pathogenesis of contact dermatitis^15–17^. In murine contact hypersensitivity (CHS), neutrophils are important in the elicitation phase, when neutrophil recruitment to the hapten-challenged site leads to infiltration of hapten-specific CD8 + T cells and development of a CHS response^18–20^. Furthermore, more recent studies described a requirement of neutrophils for both the sensitisation and elicitation phases of CHS^21–23^. However, the detailed role of neutrophils in the CHS model remains unknown. Since loss-of-function *IL36RN* mutations cause significant neutrophil infiltration in skin lesions, we hypothesised that *Il36rn* mutations could be one of the exacerbating factors in CHS. Therefore, to clarify these questions, we examined the CHS response in *Il36rn*^−/−^ mice.

## Results

### Estimation of ear thickness and histological characteristics of *Il36rn*^−/−^ mice after DNFB challenge

**Figure 1:**
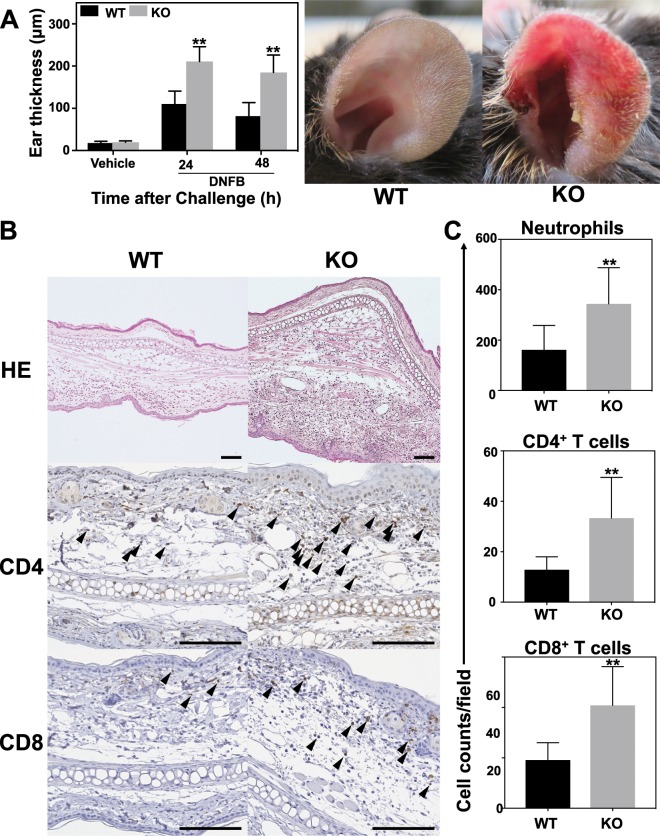
*Il36rn*^−/−^ mice have increased contact hypersensitivity. (**A**) Left: Quantification of ear thickness in wild-type and *Il36rn*^−/−^ mice 24 h and 48 h after DNFB challenge (n = 26 mice; **p < 0.01 versus wild-type mice). Right: Representative images of wild-type and *Il36rn*^−/−^ mice ears shown at 48 h after DNFB challenge (WT: wild-type mice, KO: *Il36rn*^−/−^ mice). (**B**) Representative histological and immunohistochemical images of ear skin sections from wild-type and *Il36rn*^−/−^ mice 48 h after challenge with DNFB. Scale bars, 100 µm. Positive CD4 + T cells and CD8 + T cells are indicated by black arrows. (**C**) Cell counts of neutrophils, CD4 + , and CD8 + T cells/high power field (HPF) in ear cross-sections from wild-type and *Il36rn*^−/−^ mice. Cell counts were performed at 100x magnification in 12 sections per mouse (n = 26 mice; **p < 0.01 versus wild-type mice).

Ear thickness was evaluated 24 h and 48 h after 1-fluoro-2,4-dinitorobenzene (DNFB) challenge in wild-type and *Il36rn*^−/−^ mice to test if a deficiency in IL-36Ra affects the CHS response. Interestingly, the *Il36rn*^−/−^ mice showed a significant increase in ear thickness compared with the wild-type mice at both 24 h (212%; **p < 0.01) and 48 h (323%; **p < 0.01) after DNFB challenge (Fig. 1A). Histopathologically, *Il36rn*^−/−^ mice presented prominent oedema and infiltration of inflammatory cells as compared with wild-type mice (Fig. 1B). We assessed the infiltration of neutrophils (161.5 ± 14.02 (WT) and 344.3 ± 20.71 (*Il36rn*^−/−^) cells/field, **p < 0.01), CD4 + T-cells (12.83 ± 1.58 (WT) and 33.42 ± 4.978 (*Il36rn*^−/−^) cells/field, **p < 0.01), and CD8 + T-cells (19.08 ± 2.098 (WT) and 41 ± 4.723 (*Il36rn*^−/−^) cells/field, **p < 0.01) (Fig. 1C). Our results suggested a significant increase in the inflammatory cells in *Il36rn*^−/−^ mice as compared to that in wild type mice. Thus, an IL-36Ra deficiency increases infiltration of inflammatory cells during the CHS response.

### Cytokine and chemokine expression in ear tissue of *Il36rn*^−/−^ mice

**Figure 2:**
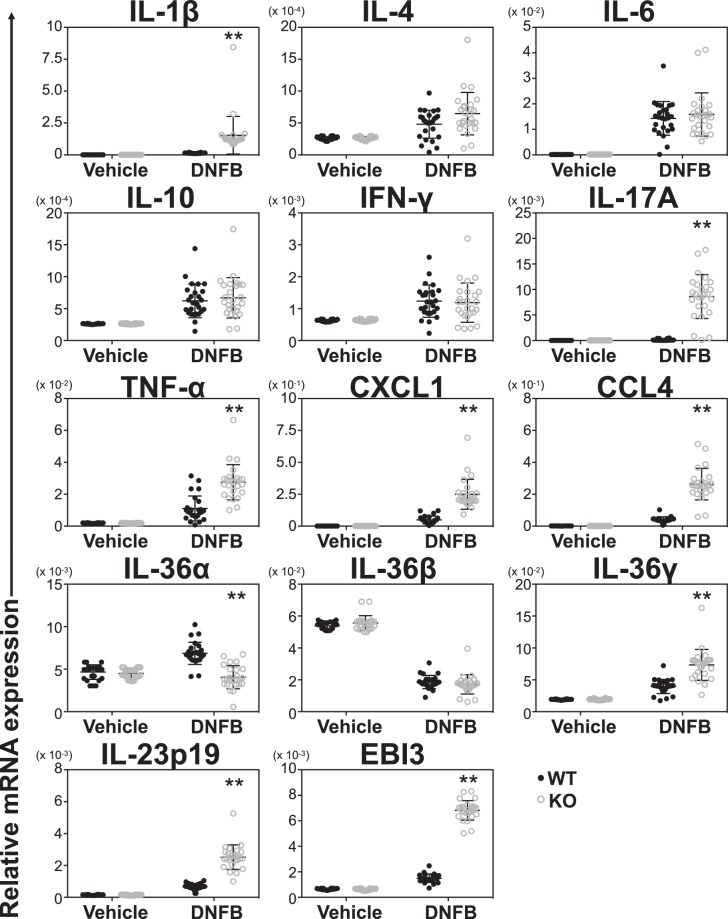
Cytokine expression is increased by IL36RN deficiency. Expression of IL-1β, IL-4, IL-6, IL-10, IFN-γ, IL-17A, TNF-α, CXCL1, IL-36α, IL-36β, IL-36γ, IL-23p19, and EBI3 mRNA. There was increased IL-1β, IL-17A, TNF-α, CXCL1, IL-36α, IL-36γ, IL-23p19, and EBI3 mRNA expression in *Il36rn*^−/−^ mice compared with that in wild-type (n = 26 mice; **p < 0.01 versus wild-type mice). GAPDH mRNA was used as an internal control.

Expression of IL-1β, IL-4, IL-6, IL-10, interferon (IFN)-γ, IL-17A, tumor necrosis factor (TNF)-α, C-X-C motif chemokine ligand (CXCL)1, CC chemokine ligand (CCL)4, IL-36α, IL-36β, IL-36γ, IL-23p19, and Epstein-Barr virus induced gene 3 (EBI3) in ear tissue at 24 h after a DNFB challenge was examined by real-time polymerase chain reaction (RT-PCR) in *Il36rn*^−/−^ and wild-type mice (Fig. 2). *Il36rn*^−/−^ mice had increased expression levels of IL-1β, IL-17A, TNF-α, CXCL1, CCL4, IL-36γ, IL-23p19, and EBI3 relative to their expression in wild-type mice. Although levels of IL-4 in *Il36rn*^−/−^ mice tended to be higher than those in wild-type mice, these differences were not statistically significant. By contrast, mRNA levels of IL-36α decreased in *Il36rn*^−/−^ mice and the loss of IL-36Ra did not affect IL-6, IL-10, IFN-γ, and IL-36β mRNA expression in comparison to wild-type expression levels. Thus, IL-36Ra deficiency alters the expression of different cytokines, contributing to the CHS response observed in the loss of function mutants.

### Effect of TAK-242 on CHS response

The wild-type mice showed an increase in neutrophil infiltration more than the *Il36rn*^−/−^ mice, indicating that, acquired immunity plays a central role in the CHS response, but we speculated that the innate immune system plays a key role in enhancing the contact dermatitis response in *Il36rn*^−/−^ mice. A previous study reported that TAK-242 administration suppressed neutrophil infiltration in an imiquimod-induced psoriasis model^8^, so we considered that suppressing the innate immune system and infiltration of neutrophils by suppressing downstream of TLR4 could suppress this CHS response even in the CHS model. Thus, we performed a treatment experiment using TAK-242, which, selectively inhibits TLR4.

**Figure 3:**
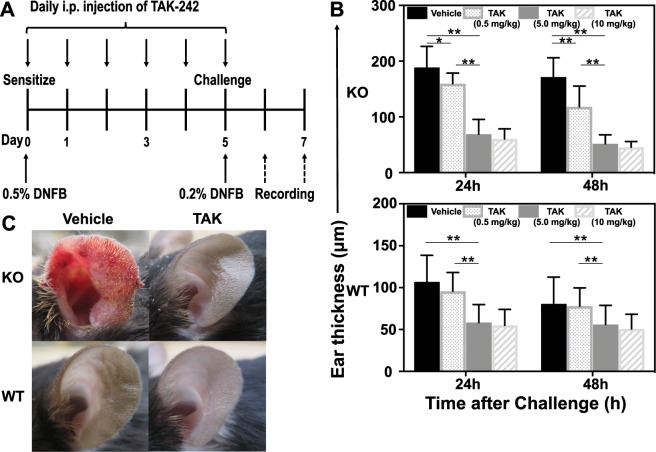
Intraperitoneal administration of TAK-242 decreases contact hypersensitivity in both wild-type and *Il36rn*^−/−^ mice. (**A**) Protocols for the development of DNFB-induced contact hypersensitivity. For the prevention experiments, both wild-type and *Il36rn*^−/−^ mice were treated with intraperitoneal (i.p.) injection of TAK-242 (0.5, 5.0, and 10 mg/kg) on days 0–5. (**B**) Top: Quantification of ear thickness in *Il36rn*^−/−^ mice administered TAK-242 (0.5, 5.0, or 10 mg/kg) or vehicle 24 h and 48 h after DNFB challenge (n = 26 mice; *p < 0.05, **p < 0.01). Bottom: Quantification of ear thickness in wild-type mice administered TAK-242 (0.5, 5.0, or 10 mg/kg) or vehicle 24 h and 48 h after DNFB challenge (n = 26 mice; **p < 0.01). (TAK: TAK-242). (**C**) Representative images of wild-type and *Il36rn*^−/−^ mouse ears administered TAK-242 (5.0 mg/kg) or vehicle 48 h after DNFB challenge.

We examined the effect of the TLR4 inhibitor, TAK-242, on the elevated CHS response observed in *Il36rn*^−/−^ mice. TAK-242 (0.5, 5.0, or 10 mg/kg/day) or the same amount of vehicle was intraperitoneally administered for 6 days (day 0–5) before DNFB challenge and CHS response was assessed in the different mice lines (Fig. 3A).

CHS response at 24 and 48 h in *Il36rn*^−/−^ mice (24 h; 15.48% decrease; *p < 0.05, 48 h; 31.17% decrease; **p < 0.01) was reduced by administration of TAK-242 (0.5 mg/kg/day) as compared to the vehicle control (Fig. 3B). CHS response at 24 and 48 h in wild-type mice was not significantly different between TAK-242 administration (0.5 mg/kg/day) and the vehicle control. CHS response at 24 and 48 h in *Il36rn*^−/−^ mice (24 h; 63.34% decrease; **p < 0.01, 48 h; 69.73% decrease; **p < 0.01) and wild-type mice (24 h; 45.32% decrease; **p < 0.01, 48 h; 30.48% decrease; **p < 0.01) was reduced by TAK-242 administration (5.0 mg/kg/day) as compared to the vehicle control (Fig. 3B,C). Moreover, CHS response at 24 and 48 h in *Il36rn*^−/−^ and wild-type mice was not significantly different between 10 mg/kg/day TAK-242 and 5.0 mg/kg/day TAK-242. In addition, administration of TAK-242 (5.0 mg/kg/day) after DNFB challenge (days 5 and 6) showed only focal inhibitory CHS responses in wild-type and *Il36rn*^−/−^ mice (data not shown).

Thus, administration of TAK-242 before DNFB challenge diminishes CHS responses in mice with or without IL-36Ra deficiency. Furthermore, TAK-242 showed a dose-dependent effect up to 5.0 mg/kg/day. However, there was no significant difference in the effect between TAK-242 doses of 5.0 mg/kg/day and 10 mg/kg/day.

### Effect of TAK-242 on inflammatory cell recruitment

**Figure 4:**
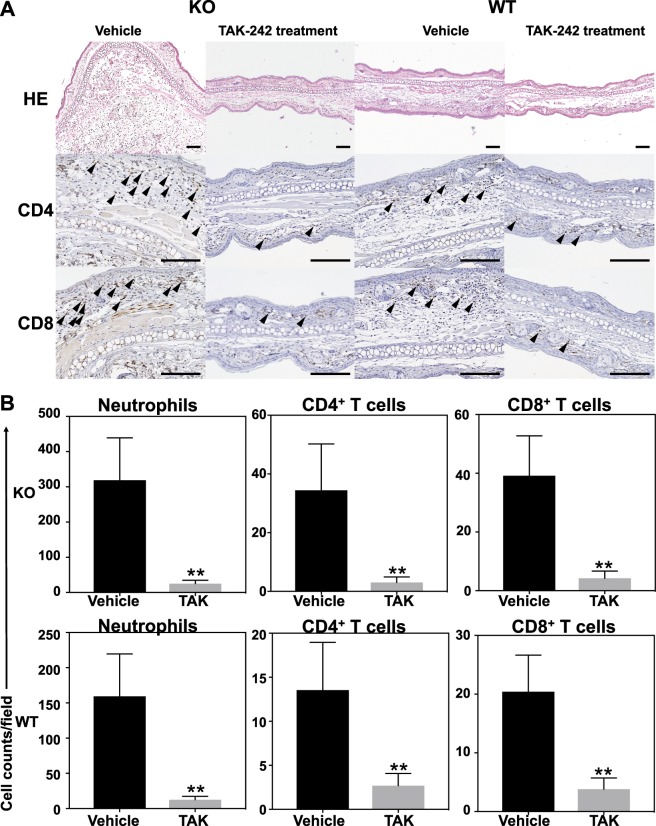
Intraperitoneally administered TAK-242 suppresses inflammatory cell infiltration. (**A**) Representative histology and immunohistochemical sections of ear skin from *Il36rn*^−/−^ and wild-type mice administered TAK-242 (5.0 mg/kg) or vehicle 48 h after DNFB challenge. Scale bars, 100 µm. (**B**) Top: Cell counts of neutrophils and CD4 + and CD8 + T cells/high power field (HPF) in ear cross-sections of *Il36rn*^−/−^ mice administered TAK-242 (5.0 mg/kg) or vehicle (n = 26 mice; **p < 0.01 versus vehicle control). Bottom: Cell counts of neutrophils and CD4 + and CD8 + T cells/high power field (HPF) in ear cross-sections of wild-type mice administered TAK-242 (5.0 mg/kg) or vehicle (n = 26 mice; **p < 0.01 versus vehicle control). Cell counts were performed at 100x magnification in 12 sections per mouse. (TAK: TAK-242).

Given that TAK-242 (5.0 mg/kg/day) had an effect on the CHS response in the mouse model, we assessed its effect on inflammatory cell recruitment. Inflammatory cell recruitment was decreased by TAK-242 (5.0 mg/kg/day) administration in both wild-type and *Il36rn*^−/−^ mice. In our histological analysis, the numbers of neutrophils were decreased in both wild-type (133.3 ± 25.36 (Vehicle) vs. 20.92 ± 2.781 (TAK) cells/field, **p < 0.01) and *Il36rn*^−/−^ mice (293 ± 38.9 (Vehicle) vs. 24.67 ± 2.993 (TAK) cells/field, **p < 0.01) (Fig. 4A,B). In our immunohistological analysis, the numbers of CD4 + and CD8 + T cells were significantly decreased by TAK-242 (5.0 mg/kg/day) in both wild-type and *Il36rn*^−/−^ mice (Fig. 4A). The number of CD4 + T cells were decreased in both wild-type (13.67 ± 1.662 (Vehicle) vs. 2.667 ± 0.4323 (TAK) cells/field, **p < 0.01) and *Il36rn*^−/−^ mice (34.58 ± 4.853 (Vehicle) vs. 2.917 ± 0.5833 (TAK) cells/field, **p < 0.01) (Fig. 4B). In addition, the numbers of CD8 + T cells were decreased in both wild-type (20.25 ± 1.903 (Vehicle) vs. 3.833 ± 0.5882 (TAK) cells/field, **p < 0.01) and *Il36rn*^−/−^ mice (39.08 ± 4.188 (Vehicle) vs. 4.167 ± 0.747 (TAK) cells/field, **p < 0.01) (Fig. 4B).

CHS response and the number of inflammatory cells were similar in TAK-242-treated wild-type and *Il36rn*^−/−^ mice. Thus, increased CHS response and inflammatory cell recruitment was completely inhibited by TAK-242 treatment, regardless of the genetic background.

### TAK-242 treatment changes cytokine and chemokine mRNA expression in mouse ear tissue

**Figure 5:**
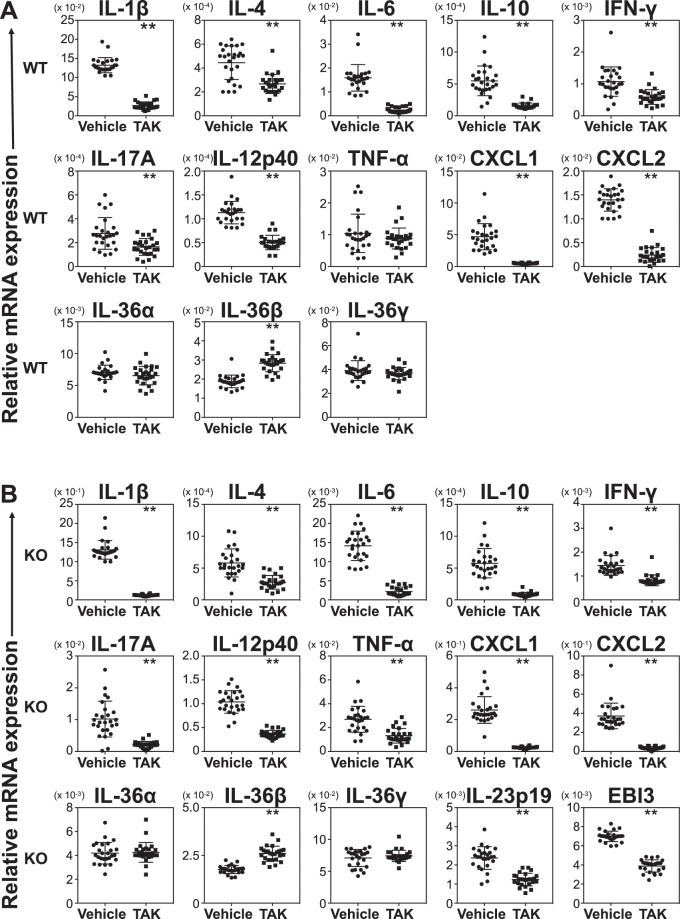
Cytokine expression in wild-type and *Il36rn*^−/−^ mice administered TAK-242. (**A**) There was a decrease in IL-1β, IL-4, IL-6, IL-10, IFN-γ, IL-17A, IL-12p40, CXCL1, and CXCL2 mRNA levels, but an increase in IL-36β mRNA in wild-type mice treated with TAK-242 as compared with vehicle control (n = 26 mice; **p < 0.01, versus vehicle control). (TAK: TAK-242). (**B**) There was a decrease in IL-1β, IL-4, IL-6, IL-10, IFN-γ, IL-17A, IL-12p40, TNF-α, CXCL1, CXCL2, IL-23p19, and EBI3 mRNA, but an increase in IL-36β mRNA in *Il36rn*^−/−^ mice treated with TAK-242 as compared with vehicle control (n = 26 mice; **p < 0.01 versus the vehicle control).

To assess how TAK-242 inhibits CHS responses and inflammation, we analysed cytokine and chemokine mRNA levels in ear tissue in wild-type and *Il36rn*^−/−^ mice, with and without TAK-242 treatment. In wild-type mice, the mRNA expression levels of IL-1β, IL-4, IL-6, IL-10, IFN-γ, IL-17A, IL-12p40, CXCL1, and CXCL2 were significantly reduced by TAK-242 treatment (Fig. 5A). In contrast, IL-36β mRNA expression significantly increased after TAK-242 treatment (Fig. 5A). There were no significant differences in the mRNA expression levels of TNF-α, IL-36α, and IL-36γ between the TAK-242 treatment and untreated groups (Fig. 5A). In *Il36rn*^−/−^ mice, mRNA expression levels of IL-1β, IL-4, IL-6, IL-10, IFN-γ, IL-17A, IL-12p40, TNF-α, CXCL1, CXCL2, IL-23p19, and EBI3 were significantly reduced by TAK-242 treatment, while IL-36β expression significantly increased (Fig. 5B). There were no significant differences in the expression levels of IL-36α and IL-36γ in the TAK-242 treatment and control groups (Fig. 5B). Thus, TAK-242 treatment decreased mRNA expression levels of Th1, Th2, Th17 cytokines, proinflammatory cytokines, and chemokines, while IL-36β mRNA levels increased. In addition, IL-23p19 and EBI3 mRNA levels were also decreased by TAK-242 treatment in *Il36rn*^−/−^ mice.

## Discussion

The results of our study demonstrate that the CHS response is enhanced in *Il36rn*^−/−^ mice.

The results of histopathological examination showed that the number of neutrophils and lymphocytes in the CHS lesion sites significantly increased in *Il36rn*^−/−^ mice compared to that in wild-type mice. Moreover, expression of cytokines and chemokines in the CHS lesions revealed that IL-1β, IL-17A, TNF-α, CXCL1, CCL4, IL-36γ, IL-23p19, and EBI3 significantly increased in *Il36rn*^−/−^ mice compared with that in wild-type mice. The treatment experiments with TAK-242, which has proven safe for human use^8,24^, showed a decrease in the CHS response in both wild-type and *Il36rn*^−/−^ mice. Furthermore, it was revealed that in treated CHS lesion, the expression of cytokines and chemokines such as IL-1β, IL-4, IL-6, IL-10, IFN-α IL-17A, IL-12p40, CXCL1, and CXCL2 decreased in both wild-type and *Il36rn*^−/−^ mice compared with levels in the vehicle control. However, there were no significant differences in the expression levels of IL-36α between vehicle control and treated group in both wild-type and *Il36rn*^−/−^ mice, consistent with a previous report that IL-36α is not essential for induction of local inflammation during DNFB-induced CHS^25^. Collectively, the results of this study suggest that mutations in *Il36rn* enhance the CHS response by acting on various cytokines and chemokines involved in neutrophil migration, and that inhibiting TLR4 is likely to affect the production of these cytokines and chemokines.

The CHS response was enhanced in *Il36rn*^−/−^ mice, and we considered that this is attributable to the increase in neutrophils. Actually, several recent studies have reported that neutrophils play an important role in both the sensitization and elicitation phases of contact dermatitis^21–23^. When there is a deficiency in IL-36Ra function, the signal transduction continues via IL-36R. Because IL-36R induces the production of inflammatory cytokines by activating NF-κB and MAPK^9,10^, IL-1β, TNF-α, and IL-36γ are increased in the CHS lesion and result in the sustained activation of the receptor. The increase in TNF-α upregulates the production of IL-17A from Th17 cells, which subsequently increases chemokines such as CXCL1 and CXCL2^26–29^. In fact, it has been reported that IL-17A regulates epidermal keratinocytes to produce chemokines such as CXCL1 and CXCL2 to induce neutrophils. In addition, it has also been reported that IL-39, which is a hetero dimer of IL-23p19 and EBI3, is downstream of IL-36γ and involved in the induction of neutrophils^30^. When IL-36Ra is lost, neutrophil-induced pathways, triggered by increased chemokines and IL-39, are further amplified through a positive feedback loop, which is consistent with the increase in neutrophils which we observed in our histopathological investigations.

We considered that TAK-242 regulated both the sensitisation and elicitation phases of contact dermatitis by inhibiting the CHS response. In the sensitisation phase, the CHS response can be suppressed by inhibiting the activation of the innate immune system^31^. In this study, the gene expression of cytokines such as IL-6, IFN-γ, IL-4, IL-17, and IL-10 was reduced in the CHS lesions of both wild-type and *Il36rn*^−/−^ mice after TAK-242 treatment. Other studies have shown that these cytokines are produced by macrophages and effector T cells^32,33^, and that TLR4 increases the sensitivity of contact dermatitis. TLR4 is expressed in several non-immune cells such as keratinocytes and sebocytes, and antigen-presenting cells such as macrophages and dendritic cells (DCs)^33–35^, and is required for the activation of the innate immune system, which is necessary for the sensitization of allergens^34,36–38^. When the innate immune system is activated, the migration of dermal DCs to regional lymph nodes, which is the sensitisation phase of contact dermatitis, is initiated. Thus, if the sensitisation phase is inhibited by TAK-242, CHS is reduced in both wild-type and *Il36rn*^−/−^ mice.

In the induction phase of contact dermatitis, the CHS response can be inhibited primarily by suppressing the activation of both effector T cells and TNF and iNOS-producing DCs (Tip-DC)^31^. By suppressing the activation of effector T cells, the production of IL-17A in Th17 cells decreases, followed by a decrease in the production of CXCL1 and CXCL2 chemokines. In fact, the mRNA expression of IL-17A, CXCL1, and CXCL2 decreased in CHS lesions of both wild-type and *Il36rn*^−/−^ mice in this experiment. Histopathologically, the number of neutrophils in tissues reduced, reflecting a decrease in the expression of CXCL1 and CXCL2.

Dysfunction of IL-36Ra upregulates inflammatory cytokines such as TNF-α. Subsequently, these cytokines regulate Tip-DCs and promote IL-17A infiltration. The decrease in IL-36R signalling can be explained by the decrease in the expression of IL-39 downstream of IL-36γ. We found that the expression of TNF-α in the CHS lesions of *Il36rn*^−/−^ mice increased. Furthermore, the present study showed that a decrease, in TNF-α expression levels was observed only in *Il36rn*^−/−^ mice treated with TAK-242. Moreover, it has been reported that overexpression of IL-36 cytokines in allergic contact dermatitis patients can induce Th17 cytokines^39^. From these reasons, we considered this to be one of the characteristics of *Il36rn* deficiency. Thus, we concluded that TAK-242 blocks TNF-α induction by inhibiting TLR4 expression on the cell surface of Tip-DCs and it suppresses effector T cell activation.

In summary, this study demonstrates that the activation and intensity of CHS response depend on the deficiency of IL-36Ra. Furthermore, we demonstrated that blocking TLR4 function with TAK-242 inhibits the CHS response in both *Il36rn*^−/−^ and wild-type mice. The *Il36rn* mutation increased the CHS response by eliciting excessive infiltration of neutrophils into the skin, which was due to the activation of IL-36 receptor-mediated sustained inflammatory signalling. These results suggest that a deficiency in IL-36Ra intensifies the CHS response and that blocking TLR4 signals by TAK-242 is a promising therapeutic strategy for treating contact dermatitis.

## Materials and Methods

### Ethics statement

The mice were handled ethically according to the Regulations for the Management of Laboratory Animals at Fujita Health University. The experimental protocol for the ethical use of these animals was approved by the Animal Care and Use Committee at Fujita Health University (Permit No.: AP16079).

### Mice

Gender matched female wild-type (C57BL/6NCr1) and *Il36rn*^−/−^ mice (Aged 6-12w) were used for all experiments. *Il36rn*^−/−^ mice were generated as previously reported^8^ and genotypically confirmed by allele-specific PCR. Control C57BL/6NCr1 animals were obtained from Charles River Laboratories (Charles River Laboratories, Inc., Wilmington, Massachusetts, USA). All experiments were repeated thrice using healthy and fertile mice that did not display any evidence of infection or disease. All mice were housed in a specific pathogen-free barrier facility and screened regularly for pathogens.

### Induction of contact hypersensitivity

The CHS mouse model was induced with DNFB (Wako Pure Chemicals, Tokyo, Japan) as previously reported^40^. Briefly, age-matched mice were sensitised with 25 µl 0.5% DNFB in acetone/olive oil (4:1) on a shaved back on day 0. On day 5, sensitised mice were topically challenged with 15 µl 0.2% DNFB in acetone/olive oil (4:1) on each side of both ears. Ear thickness was measured with dial thickness gauges (Peacock, Ozaki MFG. CO., Ltd, Chiba, Japan) before DNFB challenge and 24 h and 48 h after DNFB challenge. Each ear lobe was measured three times and the mean of those values was used for analysis.

### Histological analysis of ear sections

Mice ears were harvested 48 h after DNFB challenge; central strips of the ears were fixed in 3.5% paraformaldehyde and embedded in paraffin. From these preparations, 6-µm paraffin sections were stained with haematoxylin and eosin (H&E) for conventional histological evaluation. Dermal neutrophil infiltration was evaluated by counting the number of neutrophils present in 12 high-power fields (0.07 mm^2^). Each section was examined independently by two investigators in a blind study and the mean of their measurements was used for analysis.

### Immunohistochemical staining

Paraffin-embedded tissues were cut into 6-µm sections, deparaffinised in xylene, and rehydrated in phosphate buffered saline (PBS). Deparaffinised sections were treated with endogenous peroxidase blocking solution (horse serum diluted 1:1 in buffer: PBS + bovine serum albumin 1%) for 15 min at room temperature. Sections were then incubated overnight at 4 °C in rat monoclonal antibodies (mAb) specific for CD4 (clone: D7D2Z; catalogue number: #25229; Cell Signalling Technology, Tokyo, Japan; dilution 1:300) and CD8 (clone: D4W2Z; catalogue number: #98941; Cell Signalling Technology, Tokyo, Japan; dilution 1:1000). Sections were then washed in PBS buffer and biotin-conjugated secondary antibodies were then applied followed by incubation with avidin-biotin complex (Vector Laboratories: VECTASTAIN Elite ABC Kit #PK-6101) for 30 min at room temperature followed by three washes with PBS for 15 min each. Peroxidase activity was observed using an ImmPACT DAB Substrate Kit (Vector Laboratories: #SK-4105) and samples were counterstained with haematoxylin. For negative control, primary antibody was not added to the sections.

### RNA isolation and RT-PCR

Total RNA was extracted from ear tissue samples using Qiagen RNeasy spin columns (QIAGEN, Crawley, UK) and subsequently reverse transcribed to cDNA using the Prime Script RT Reagent Kit (Takara Bio INC, Shiga, Japan). Expression levels of IL-1β (Mm.PT.58.41616450), IL-4 (Mm.PT.58.32703659), IL-6 (Mm.PT.58.10005566), IL-10 (Mm.PT.58.13531087), IL-17A (Mm.PT.58.6531092), IL-12p40 (Mm.PT.58.1240997), IL-36α (Mm.PT.58.12651602), IL-36β (Mm.PT.58.11528127), IL-36γ (Mm.PT.58.30810984), CXCL1 (Mm.PT.58.42076891), CCL4 (Mm.PT.58.5219433), CXCL2 (Mm.PT.58.1045839), IFN-γ (Mm.PT.58.41769240), TNF-α (Mm.PT.58.12575861), IL-23p19 (Mm.PT.58.41340226), and EBI3 (Mm.PT.58.45979145) were measured by RT-PCR using the Light Cycler System (F. Hoffmann-La Roche, Ltd, Basel, Switzerland). The PCR samples were set up in microcapillary tubes as a 20 µl reaction consisting of 2.0 µl diluted cDNA solution, and the PCR program was set according to the manufacturer’s instructions. Glyceraldehyde-3-phosphate (GAPDH; Mm.PT.39a.1) was used as an internal control. Relative mRNA expression levels of different target genes normalised to GAPDH expression were calculated using the 2-∆∆Ct method. Primer sequences used for each gene were selected from pre-validated PrimeTime qPCR Assays (Integrated DNA Technologies, Coralville, Iowa, USA).

### TLR4 inhibition with TAK-242

Wild-type and *Il36rn*^−/−^ mice were treated with an intraperitoneal injection of TAK-242 (0.5, 5.0, or 10 mg/kg) (catalogue number: CS-0408; Chemscene, Monmouth Junction, NJ, USA) or vehicle (DMSO solution) on days 5–6 or days 0–5. Ear thickness was measured with dial thickness gauges before DNFB challenge (day 5), 24 h (day 6), and 48 h (day7) after DNFB challenge. In addition, ear tissue samples were collected for RT-PCR and histopathological analysis.

### Statistical analysis

Data were analysed with GRAPHPAD PRISM software (version 7; GraphPad Software, La Jolla, CA, USA) and presented as means ± SD. Mann-Whitney U test or one-way analysis of variance (ANOVA) was used to determine the statistical significance of differences. Values of *p* < 0.05 were defined as significant.
